# Unplanned pregnancy after ultrasound-guided percutaneous microwave ablation of uterine fibroids: A follow-up study

**DOI:** 10.1038/srep18924

**Published:** 2016-01-06

**Authors:** Zhang Bing-song, Zhang Jing, Han Zhi-yu, Xu Chang-tao, Xu Rui-fang, Li Xiu-mei, Liu Hui

**Affiliations:** 1 Department of Interventional Ultrasound, Chinese PLA General Hospital, 28 Fuxing Road, Beijing 100853 China.

## Abstract

A follow-up study was performed with 169 women of childbearing age who underwent ultrasound-guided percutaneous microwave ablation (UPMWA) therapy for symptomatic uterine fibroids in the Chinese PLA General Hospital from June 2007 to December 2014. This study aimed to observe the incidence of unplanned pregnancies in these women after UPMWA treatment in order to evaluate its effect on natural conception. Ten unplanned pregnancies in nine women were occurred. Of the nine patients, six did not want the pregnancy and chose for induced abortion to end the pregnancy at an early stage. Three chose to continue with the pregnancy and gave birth to a healthy term infant delivered by cesarean section (of these three patients, two had been previously diagnosed as infertility). None of the patients had any serious obstetric complications. After UPMWA treatment for uterine fibroids, patients may conceive naturally, the impact of the procedure on fertility and pregnancy outcomes is worthy of further prospective study in larger sample.

Uterine fibroids are the most common benign tumors encountered in women of reproductive age. Associated symptoms include dysmenorrhea, spotting, hypermenorrhea leading to anemia, lower abdominal pain, infertility, pressure on adjacent organs, and disorders of micturition and defecation^1^. The reported incidence of fibroids ranges from 5.4% to 77.0%^2^, and with the current tendency to delay childbearing until later in life this prevalence is likely to increase in nulliparous women^3^. The presence of uterine fibroids has a particularly important impact on women who may desire to become pregnant, as fibroids can impair fertility and cause specific complications in pregnancy^4^. Thus, treatment goals include not only alleviation of the symptoms, but also reservation or even amendment of the ability of fertility. Recently, ultrasound-guided percutaneous microwave ablation (UPMWA) has been widely used to treat symptomatic myomas and adenomyosis^5^,^6^,^7^,^8^,^9^,^10^,^11^. This has been shown to be a feasible and safe approach for treating symptomatic uterine fibroids; moreover, the procedure is easy, fast, and minimally invasive. UPMWA appears to be an optional therapy for patients with symptomatic uterine fibroids who want to keep their uterus intact. Nonetheless, the effect of UMPWA on fertility has not been revealed; because of the novel nature of the procedure, it cannot be used to study its effect on fertility in patients who have reproductive requirements; therefore, initial UPMWA trials were restricted to patients with no desire for fertility in the future. The present study aimed to observe unplanned pregnancies that occurred after treatment of uterine fibroids using UPMWA, in order to accumulate evidence to evaluate its effect on natural conception.

## Materials and Methods

### Patients

This study included patients of childbearing age who had undergone UPMWA treatment for uterine fibroids at our hospital between June 2007 and December 2014. The following inclusion criteria were applied: all patients had been diagnosed with uterine fibroids using ultrasonography and contrast-enhanced MRI (ceMRI) at our hospital; they had one of the following symptoms: menorrhagia or metrorrhagia, pelvic pain, bulk pressure, or frequent urination; patients had either completed childbearing or no longer desired fertility. The patients sought treatment and rejected hysterectomy and myomectomy. Patients with lack of an appropriate percutaneous access route, history of malignancy, abnormal ThinPrep cytology test results, pelvic infection, or contraindications for intravenous anesthesia were excluded from the study. The diagnosis was confirmed by histopathological examination of specimen obtained from ultrasound guided percutaneous biopsy before treatment. The study was carried out in accordance with the protocol approved by the Institutional Review Board of PLA General Hospital (ratification no. 20100930-004, registration no. ChiCTR-TRC-10001119). Each woman received written information about the study, and informed consent was obtained from all subjects.

### Instruments

A microwave tumor coagulator (KY-2000 MW; Kangyou Medical Instruments Co., Nanjing, China), with a frequency of 2450 MHz. The needle antenna has a 15-gauge external diameter (1.9 mm) and was 18 cm in length. The distance from the aperture of the microwave emission to the needle tip was 11 mm, and the emission aperture was 1 mm.

An Acuson Sequoia 512 Ultrasound System (Signature 10.2; Siemens Medical Solutions, Inc., Mountain View, CA) Siemens Sequoia 512 Computer Color ultrasonograph with a puncture-guided device and low MI contrast-enhanced function was used. The frequency of the probe was 2.5–4.5 MHz.

### Treatment procedure

The UPMWA treatment protocol was based on previously published protocols^5^,^6^,^7^,^8^,^9^,^10^,^11^. In brief, ablation was performed under conscious intravenous sedation (intravenous infusion of flurbiprofen ester 2 mg/kg, supplemented by pumping 4 mg/(kg·h)^–1^ propofol). The patients were placed in a supine position. The microwave antenna was inserted into the fibroids under ultrasound guidance. Based on the dose-effect relationship of microwave ablation, the output power was set at 50 or 60 W. During microwave emission, the ablation area was monitored by ultrasound in real time. Contrast-enhanced ultrasound (SonoVue, Bracco SinePharm) was performed immediately after the procedure for preliminary evaluation of ablation efficacy, and if blood stream perfusion was detected in the fibroid, a supplementary treatment was performed. Before the treatment, fibroid volume was assessed using ultrasonography. The mean diameter and volume of the myomas were calculated via ultrasound using the following formulae: Mean diameter = (length + width + height)/3, and volume = 4/3 × π × r^3^, where r is the mean radius at ultrasound (mean diameter/2). One day after the treatment, CEUS was performed to evaluate the ablation volume rate, which was calculated as the non-perfused volume (NPV)/targeted fibroid volume × 100%.

### Study method

All unplanned pregnancies of the patients after UPMWA were followed up and recorded. The pregnant patients were then analyzed for various parameters, including their general condition (such as weight, body mass index (BMI, calculated as weight in kilograms divided by the square of height in meters), and age); childbearing history; volume, location, and type of FIGO classification^12^ of the fibroids before therapy; ablation rate of the fibroids; discharge of necrotic tissues; interval between UPMWA therapy and pregnancy; pregnancy outcome, pregnancy process, and neonatal outcome; and adverse effects.

Statistical analyses were performed using commercially available software (SPSS version 13.0; SPSS, Inc., Chicago, IL). All quantitative data were expressed as means ± standard deviation (SD).

## Results

From June 2007 to December 2014, 169 women of childbearing age underwent UPMWA for symptomatic uterine fibroids in our hospital. All patients received a successful single-pass UPMWA treatment, without any serious complications and side effects. All the patients were recommend to use condoms for contraception after treatment, but 91 patients failed to comply with this principal strictly. There were ten unplanned pregnancies in nine women during the course of our follow-up study.

At the time of treatment, the mean age was of the nine women was 34.2 ± 3.7 (range, 27–40) years (Table 1). The mean BMI of the patients was 24.2 ± 1.5 (range, 22.1–27.1). Of the nine patients, six had already completed their families (in China, only one child is recommended per family), and opted for induced abortions to terminate their pregnancies during the early stage. Three of the patients who became pregnant after UPMWA chose to continue with their pregnancies and gave birth to healthy term infants by cesarean section. Two of these patients had been previously diagnosed with infertility.

Before treatment, menorrhagia was the most common symptom, affecting five of the nine women (5/9, 55.56%), followed by abdominal pressure, which affected three of the nine women (3/9, 33.33%). Two patients suffered from infertility (2/9, 22.22%) (Defined as inability to get pregnant for more than 12 consecutive months as reported by the patient) (Table 1). The follow-up was performed every three months after treatment, and all the clinical symptoms were significantly alleviated or disappeared gradually. Six months after the treatment, the anemia was cured in both the patients (2/2, 100%), the menorrhagia was cured in all the five patients (5/5, 100%), and the abdominal compressive symptoms improved in three of the four patients (3/4, 75%).

13 fibroids in this subgroup of women, 11 of the 13 fibroids were treated, with the others not treated owing to their small size. The mean diameter of the treated fibroids was 5.30 ± 1.03 cm (range, 4.1–7.13 cm), while the mean volume of the uterine fibroids was 86.07 ± 52.64 cm^3^ (range, 36.1–189.7 cm^3^). The treated fibroids were located in the anterior (n = 4), posterior (n = 5), and lateral (n = 2) walls of the uterus. The 11 fibroids were categorized according to the FIGO classification of uterine fibroids, and three belonged to type 3 (n = 3); three, to type 4 (n = 3); and five, type 5 (n = 5). The mean NPV ratio was 88.03% ± 14.8% (range, 55.1–98.9%). One patient experienced necrotic tissue discharge during menstruation at 5 and 6 months after treatment, and the mean diameters of the discharged necrotic tissue were 1.5 cm and 1.7 cm (Table 2).

Of the three patients who choose to continue with their pregnancies, the pregnancies occurred at 17, 14, 7 months after the UPMWA therapy respectively. These patients were strictly monitored through their pregnancies as they were considered high-risk. Guttate vaginal bleeding occurred in one woman, but this was resolved after 1–4 weeks of bed rest. All three pregnancies had no maternal or neonatal peripartum complications (Table 3). None of the patients had serious obstetric complications such as rupture of uterus and postpartum hemorrhage. The birth weight and the Apgar score of the neonate were within the normal range in all cases (Table 3). The three patients who underwent cesarean delivery resumed normal menstruation thereafter (Table 3).

## Discussion

Uterine fibroids are common in women of reproductive age, and these benign myomas may become symptomatic and can result in subfertility. Recently, the incidence of uterine fibroids was increased in nulliparous women. Changes in the age structure of patients and the requirement for a high quality of life have rendered traditional hysterectomy no longer suitable for all patients. An increasing number of patients now seek out therapies that can preserve the uterus, making them able to have future pregnancies. This demand has promoted the development of minimally invasive technologies with the aim of the uterus^13^,^14^. Currently, the common minimally invasive approaches to treat symptomatic fibroids in women who wish to become pregnant include laparoscopic myomectomy (LM), uterine artery embolization (UAE), and high-intensity focused ultrasound (HIFU). LM is regarded as the gold standard for treating fibroids; however, the randomized controlled trials shows that there is currently insufficient evidence to evaluate the role of myomectomy to improve fertility^15^. Some authors even believe that the process of treating fibroids can also lead to morbidity and subsequent impairment of fertility^16^. UAE is also widely used to treat symptomatic fibroids^17^. Although pregnancies have been reported after UAE, there is an age-related risk of ovarian failure and an increased risk of placentation problems associated with this approach^18^. HIFU is a novel technique for uterine fibroids^19^,^20^,^21^. Magnetic resonance imaging (MRI) or ultrasound imaging can be used for continuous monitoring during the procedure. Previous studies, including individual and multicenter collaborative trials, have reported successful pregnancies after MRI-guided HIFU^22^,^23^,^24^. HIFU is offered as a potential therapeutic option to treat symptomatic fibroids in women of childbearing age who may wish to have children in the future. Although HIFU is non-invasive approach with definite curative effects, it can be time consuming, especially for large and hypervascular nodules^25^. Rabinovici *et al.*^24^ reported 54 pregnancies in 51 women after MRI-guided HIFU for uterine fibroids in 13 sites in seven countries. They reported that live births occurred in 41% of pregnancies, with a 28% spontaneous abortion rate, an 11% rate of elective pregnancy termination, and 20% of the pregnancies went beyond 20 gestational weeks. However, the ablation rate of the fibroids in that study was just over 40%, and 24% of the patients received secondary treatment to treat the fibroids.

UPMWA is a new method for treating uterine fibroids *in situ*. Not only is this technique minimally invasive, ultrasound-guided in real-time, and safe, it also has a controlled scope and shape of ablation and is associated with fewer side effects, quick recovery time, and no serious complications. Our previous studies have shown that UPMWA of uterine fibroids had ablation rates up to 88.8 ± 15.8%. Moreover, after 3, 6, and 12 months of treatment, the fibroids shrank by an average of 61.8%, 78.7%, and 93.1%, the symptom of anemia alleviated or disappeared in all the patients by 98.9%^9^,^11^. Finally, and the clinical efficacy of the treatments can be sure. The real-time ultrasound guidance used in the treatment can effectively prevent damage to a large area of the endometrium. The ablation is localized at focal lesions, rendering the organizational structure outside the lesions, especially the pelvic vascular structure, safe; therefore, the treatment is not likely to affect ovarian function. After the treatment, the menstrual cycles of the patients remain normal, which also suggests that their ovarian cycles are normal.

Submucosal and intramural fibroids that distort the endometrial cavity are considered to impair fertility^26^. The submucus myomas can affect the morphology of the uterine cavity and endometrial environment, which may impede embryo implantation. The intramural myomas can distort the uterine cavity or compress of fallopian tubes, which may affect sperm transport and impede embryo implantation, thus causing infertility or spontaneous abortion. Under real-time ultrasound guidance, UPMWA can effectively control the thermal field under the membrane of uterine fibroids. The treatment can effectively coagulate the fibroids and shrink their volume. In our UPMWA treatments, we observed that necrotic tissues of the treated submucosal and intramural fibroids can be expelled from the vagina several months after therapy, and the fibroids may obviously shrink or even disappear. In this subgroup, the fibroids were all intramural, and the average ablation rate was 88.03% ± 15.8% (range, 55.05–99%), suggesting that without obvious injury to the endometrium after complete ablation of the myomas, natural conception may not be affected. One patient in our study expelled necrotic tissues 5 and 6 months after treatment, and six month after therapy, we found that the fibroid completely disappeared, the uterus returned to normal size, and it was found to be normal at 4 years after the treatment. This patient conceived 20 and 30 months after therapy respectively, and received induced abortions by choice. Another patient, who had been previously diagnosed with primary infertility that was diagnosed because of fibroids, gave birth to a healthy term infant by cesarean delivery. These findings suggest that because of the high ablation rate and the recovery of morphology of the uterine cavity, UPMWA treatment of submucosal and intramural fibroids may help patients diagnosed with infertility due to distorted uterine cavity or compressed fallopian tubes caused by fibroids improve their ability to conceive.

Due to the novel nature of this procedure and in accordance with the ethics requirements, initial UPMWA trials were restricted to patients with no desire for future fertility, and most of the patients were over 40 years old. With more and more cases being treated, the curative effect of UPMWA was thoroughly confirmed. Due to the age range of the patients in this study, a small percentage of the patients became pregnant. Therefore, younger patients without a strong reproductive need should be recruited for further investigation.

Because of the age factor and China’s family planning policy, only a limited number of patients chose to continue with the pregnancy after conception; the choice of continuity or termination of the pregnancy depended on social or obstetric factors but not the UPMWA procedure. This is not conducive to an objective assessment of pregnancy outcome after UPMWA. In China, the rate of cesarean delivery is close to 50%, mostly because of the prevalence of fear of pain during childbirth and poorer understanding of the risks associated with cesarean delivery in patients. Because all pregnant women in the present study opted for delivery via cesarean section, we could not investigate normal vaginal birth after UPMWA in this study. However, the existing number of cases strongly suggest that after UPMWA treatment for uterine fibroids patients may conceive naturally, and higher ablation rates may have the potential to improve the ability of infertile patients to conceive. Our preliminary results provide the possibility of prospective controlled and randomized clinical tests on patients with fertility requirements learn more about fertility and outcomes after UPMWA. As our knowledge is currently limited, further pregnancies after UPMWA treatment need to be followed up carefully, with ultrasonic evaluation of the placental site and evaluation of their placental status during pregnancy to ensure appropriate care if abnormalities are detected.

## In Conclusion

After UPMWA treatment for uterine fibroids, patients may conceive naturally, the impact of the procedure on fertility and pregnancy outcomes is worthy of further prospective study in larger sample.

## Additional Information

**How to cite this article**: Bing-song, Z. *et al.* Unplanned pregnancy after ultrasound-guided percutaneous microwave ablation of uterine fibroids: A follow-up study. *Sci. Rep.* **6**, 18924; doi: 10.1038/srep18924 (2016).

## Figures and Tables

**Table 1:** Clinical characteristics of the nine women with unplanned pregnancies after UPMWA for uterine fibroids.

Patient no.	Pregnancy no.	Age at treatment	BMI	Symptoms	Gravidity and parity at treatment	Pregnancy outcome
1	1,2	35	23.8	Menorrhagia, anemia	G2P1	Induced abortion, induced abortion
2	3	37	25	Menorrhagia, anemia	G3P1	Cesarean delivery
3	4	32	24.1	Infertility, abdominal pressure	G0P0	Cesarean delivery
4	5	33	24.4	Menorrhagia, pelvic pain	G1P1	Induced abortion
5	6	37	25.1	None	G1P1	Induced abortion
6	7	33	23.2	Menorrhagia	G1P1	Induced abortion
7	8	40	27.1	Abdominal pressure, frequent urination	G8P2	Induced abortion
8	9	34	22.1	Abdominal pressure, frequent urination	G6P2	Induced abortion
9	10	27	23.1	Infertility, menorrhagia	G1P0	Cesarean delivery

**Table 2:** Features of uterine fibroids and UPMWA therapy.

Patient no.	No. of fibroids	No. of treatments	Location of the treated fibroids	FIGO Type of the treated fibroids	Mean diameters of the treated fibroids, cm	Volume of the treated fibroid, cm^3^	Ablation rate, %	Mean diameters of discharged necrotic tissues, cm
1	1	1	P	3	5.87	105.85	80.76	1.5, 1.7
2	1	1	P	3	5.33	79.24	98.87	0
3	1	1	A	4	5.34	79.69	94.48	0
4	1	1	A	4	5.1	69.42	98.32	0
5	3	3	L, L, A	5, 5, 4	4.33, 4.37, 4.43	42.49, 43.67, 45.50	91.05, 86.88, 55.05	0
6	1	1	P	3	4.10	36.07	98.44	0
7	2	1	P	5	7.13	189.68	58.07	0
8	2	1	A	5	5.3	77.91	94.44	0
9	1	1	P	5	6.97	177.21	73.84	0

Abbreviations: P, posterior wall of the uterus; A, anterior wall of the uterus; L, lateral wall of the uterus.

**Table 3:** Pregnancy and delivery characteristics of the three women with unplanned pregnancy after UPMWA therapy for uterine fibroids who then chose to continue with their pregnancy.

Patient No.	Maternal age at delivery	Complications during pregnancy	Reason for cesarean delivery	Gestation, Weeks	Gender of neonate	Weight of neonate, kg	APGAR of neonate	Time to recover menstruation, months
2	40	Bleeding at 8 weeks of pregnancy	Social factors	40	male	2900	10	8
3	34	None	Cephalopelvic disproportion	39	male	3700	9	6
9	29	None	Social factors	39	female	3400	10	5

## References

[b1] Hoellen F., Griesinger G. & Bohlmann M. K. Therapeutic drugs in the treatment of symptomatic uterine fibroids. Expert Opin. Pharmacother. 14, 2079–2085 (2013). 23914973 10.1517/14656566.2013.825607

[b2] Okolo S. Incidence, aetiology and epidemiology of uterine fibroids. Best Pract. Res. Clin. Obstet. Gynaecol. 22, 571–588 (2008). 18534913 10.1016/j.bpobgyn.2008.04.002

[b3] Pritts E. A., Parker W. H. & Olive D. L. Fibroids and infertility: an updated systematic review of the evidence. Fertil. Steril. 91, 1215–1223 (2009). 18339376 10.1016/j.fertnstert.2008.01.051

[b4] Klatsky P. C., Tran N. D., Caughey A. B. & Fujimoto V. Y. Fibroids and reproductive outcomes: a systematic literature review from conception to delivery. Am. J. Obstet. Gynecol. 198, 357–366 (2008). 18395031 10.1016/j.ajog.2007.12.039

[b5] Zhao W. P., Han Z. Y., Zhang J. & Liang P. A retrospective comparison of microwave ablation and high intensity focused ultrasound for treating symptomatic uterine fibroids. Eur. J. Radiol. 84, 413–417 (2015). 25572326 10.1016/j.ejrad.2014.11.041

[b6] Xia M. *et al.* Research of dose-effect relationship parameters of percutaneous microwave ablation for uterine leiomyomas—a quantitative study. Sci. Rep. 4, 6469 (2014). 25267154 10.1038/srep06469 PMC4179463

[b7] Xia M. *et al.* Feasibility study on energy prediction of microwave ablation upon uterine adenomyosis and leiomyomas by MRI. Br. J. Radiol. 87, 20130770 (2014). 24947033 10.1259/bjr.20130770 PMC4112404

[b8] Lei F. *et al.* Uterine myomas treated with microwave ablation: the agreement between ablation volumes obtained from contrast-enhanced sonography and enhanced MRI. Int. J. Hyperthermia 30, 11–18 (2014). 24286236 10.3109/02656736.2013.853107

[b9] Yang Y. *et al.* Ultrasound-guided percutaneous microwave ablation for submucosal uterine fibroids. J. Minim. Invasive Gynecol. 21, 436–441 (2014). 24316137 10.1016/j.jmig.2013.11.012

[b10] Wang F. *et al.* Imaging manifestation of conventional and contrast-enhanced ultrasonography in percutaneous microwave ablation for the treatment of uterine fibroids. Eur. J. Radiol. 81, 2947–2952 (2012). 22341698 10.1016/j.ejrad.2011.12.037

[b11] Zhang J. *et al.* Ultrasound-guided percutaneous microwave ablation for symptomatic uterine fibroid treatment—a clinical study. Int. J. Hyperthermia 27, 510–516 (2011). 21756048 10.3109/02656736.2011.562872

[b12] Marret H. *et al.* Therapeutic management of uterine fibroid tumors: updated French guidelines. Eur. J. Obstet. Gynecol. Reprod. Biol. 165, 156–164 (2012). 22939241 10.1016/j.ejogrb.2012.07.030

[b13] Cheung V. Y. Sonographically guided high-intensity focused ultrasound for the management of uterine fibroids. J. Ultrasound Med. 32, 1353–1358 (2013). 23887944 10.7863/ultra.32.8.1353

[b14] Gupta J. K., Sinha A., Lumsden M. A. & Hickey M. Uterine artery embolization for symptomatic uterine fibroids. Cochrane Database Syst Rev 12, Cd005073 (2014). 25541260 10.1002/14651858.CD005073.pub4 PMC11285296

[b15] Metwally M., Cheong Y. C. & Horne A. W. Surgical treatment of fibroids for subfertility. Cochrane Database Syst Rev 11, Cd003857 (2012). 23152222 10.1002/14651858.CD003857.pub3

[b16] Iverson R. E. Jr., Chelmow D., Strohbehn K., Waldman L. & Evantash E. G. Relative morbidity of abdominal hysterectomy and myomectomy for management of uterine leiomyomas. Obstet. Gynecol. 88, 415–419 (1996). 8752251 10.1016/0029-7844(96)00218-9

[b17] Goodwin S. C. *et al.* Uterine artery embolization for treatment of leiomyomata: long-term outcomes from the FIBROID Registry. Obstet. Gynecol. 111, 22–33 (2008). 18165389 10.1097/01.AOG.0000296526.71749.c9

[b18] Sirkeci F., Narang L., Naguib N., Belli A. M. & Manyonda I. T. Uterine artery embolization for severe symptomatic fibroids: effects on fertility and symptoms. Hum. Reprod. 29, 1832–1833 (2014). 24939958 10.1093/humrep/deu147

[b19] Han N. R. & Ong C. L. Magnetic Resonance-Guided Focused Ultrasound Surgery (MRgFUS) of Uterine Fibroids in Singapore. Ann. Acad. Med. Singapore 43, 550–558 (2014). 25523859

[b20] Ren X. L. *et al.* Extracorporeal ablation of uterine fibroids with high-intensity focused ultrasound: imaging and histopathologic evaluation. J. Ultrasound Med. 26, 201–212 (2007). 17255182 10.7863/jum.2007.26.2.201

[b21] Taran F. A. *et al.* Magnetic resonance-guided focused ultrasound (MRgFUS) compared with abdominal hysterectomy for treatment of uterine leiomyomas. Ultrasound Obstet. Gynecol. 34, 572–578 (2009). 19852046 10.1002/uog.7435

[b22] Morita Y., Ito N. & Ohashi H. Pregnancy following MR-guided focused ultrasound surgery for a uterine fibroid. Int. J. Gynaecol. Obstet. 99, 56–57 (2007). 17599842 10.1016/j.ijgo.2007.03.053

[b23] Yoon S. W. *et al.* Pregnancy and natural delivery following magnetic resonance imaging-guided focused ultrasound surgery of uterine myomas. Yonsei Med. J. 51, 451–453 (2010). 20376901 10.3349/ymj.2010.51.3.451 PMC2852804

[b24] Rabinovici J. *et al.* Pregnancy outcome after magnetic resonance-guided focused ultrasound surgery (MRgFUS) for conservative treatment of uterine fibroids. Fertil. Steril. 93, 199–209 (2010). 19013566 10.1016/j.fertnstert.2008.10.001

[b25] Mikami K. *et al.* Magnetic resonance imaging-guided focused ultrasound ablation of uterine fibroids: early clinical experience. Radiat. Med. 26, 198–205 (2008). 18509719 10.1007/s11604-007-0215-6

[b26] Brady P. C., Stanic A. K. & Styer A. K. Uterine fibroids and subfertility: an update on the role of myomectomy. Curr. Opin. Obstet. Gynecol. 25, 255–259 (2013). 23562956 10.1097/GCO.0b013e3283612188

